# A Standard Reference Material for Calibration of the Cup Furnace Smoke Toxicity Method for Assessing the Acute Inhalation Toxicity of Combustion Products

**DOI:** 10.6028/jres.096.048

**Published:** 1991

**Authors:** Barbara C. Levin, Maya Paabo, Susannah B. Schiller

**Affiliations:** National Institute of Standards and Technology, Gaithersburg, MD 20899

**Keywords:** ABS, acrylonitrile-butadiene-styrene, combustion, combustion products, cup furnace, inhalation, SRM, standard reference material, toxicity tests

## Abstract

A standard reference material (SRM 1048) has been developed for use with the cup furnace smoke toxicity method. This SRM is to be used to calibrate the apparatus and to enable the user to have confidence that the method is being conducted in a correct manner and that the equipment is functioning properly. The toxicological results from this SRM should not be used to compare with those of other materials (i.e., to determine if the combustion products of a test material are more or less toxic than those from this SRM). SRM 1048 is an acrylonitrile-butadiene-styrene (ABS) and is the same as SRM 1007B which is used for calibrating the flaming mode of the Smoke Density Chamber test method (ASTM E-662 and NFPA 258). For the purposes of calibrating the cup furnace smoke toxicity method, *LC*_50_ and N-Gas values plus their respective 95% confidence limits have been determined and certified for two combustion modes (flaming and nonflaming) and two observation periods (for the 30 min exposure only and for the 30 min exposure plus a 14 d post-exposure period). The certified *LC*_50_ values plus 95% confidence intervals (in g/m^3^) are 27 ± 3 (30 min, flaming); 25 ± 3 (30 min+ 14 d, flaming); 58 ± 15 (30 min, nonflaming); and 53 + 12 (30 min+ 14 d, nonflaming). The certified N-Gas values plus 95% confidence intervals are 1.4 ± 0.2 (30 min, flaming); 1.5 ± 0.2 (30 min+ 14 d, flaming); 1.2 ± 0.2 (30 min, nonflaming); and 1.4 ± 0.2 (30 min+ 14 d, nonflaming). It is recommended that this SRM be used with the N-Gas approach to calibrate the cup furnace smoke toxicity method rather than to determine the complete *LC*_50_ values. The N-Gas approach has the advantage of providing information on the gases responsible for the lethalities as well as the toxic potency of the smoke. In addition, the N-Gas approach reduces the number of experimental animals, the time necessary to complete the calibration, and the expense.

## 1. Introduction

The United States has one of the worst fire lethality rates in the industrialized world [1]. Eighty percent of these fire deaths are caused, not by burns, but by the inhalation of toxic smoke (gases plus particulates) [2]. The past decade has seen the development of a number of methods to measure the toxic potency of the combustion atmospheres produced from the thermal decomposition of materials [3]. In the United States, two of these methods have been used more extensively than the others. The first, known as the cup furnace smoke toxicity method [4,5], was developed at the Center for Fire Research (CFR), National Bureau of Standards (NBS), now the Building and Fire Research Laboratory (BFRL), National Institute of Standards and Technology (NIST). The second was developed at the University of Pittsburgh under a grant from NIST and is called the University of Pittsburgh smoke toxicity method [6]. Neither the cup furnace smoke toxicity method nor the University of Pittsburgh smoke toxicity method have been accepted as standardized tests by ASTM or any other national or international scientific or technical society designed to develop standard test procedures. At the present time, the development of other smoke toxicity methods is still being actively pursued.

The number of smoke toxicity test apparatus users has increased. New York State has passed legislation requiring the testing of various materials before permitting their use in buildings [7]. Several other states are considering similar legislation. The test required by New York State is the one developed by the University of Pittsburgh. The U.S. Navy has recently specified the use of the cup furnace smoke toxicity apparatus for testing materials being considered for use in submarines [8]. A number of Federal agencies, industrial laboratories and testing companies are capable of conducting both test procedures.

Since the results of these smoke toxic potency tests, along with the results of other material flammability tests, are to be used in the decision making process regarding material selection and overall fire hazard, it is necessary to assure that such testing devices are installed and employed properly both by those laboratories currently conducting these tests and by new laboratories which enter the field. To help assure the reproducibility of results between laboratories, NIST has developed a SRM which can be used by all laboratories to calibrate the cup furnace smoke toxicity method. *It is important to note that this SRM was not selected to represent the toxic potency of the combustion products of an “average” material and is not designed to be used for the comparison of the relative toxic potency of the combustion products of test materials. In other words, the toxic potency of the smoke from a test material should not be compared to the toxic potency of the smoke from this SRM.*

The following criteria were used in the selection process of this SRM:

1. The material should have reproducible burning characteristics, regardless of the nature of the furnace (i.e., the material must be homogeneous and thus man-made).
2. The material should produce combustion products whose toxic potency values are within the range where the values for most materials are found.
3. Upon combustion, toxic gases in addition to CO should be generated and contribute to the lethal atmospheres.
4. The selected material should generate combustion products which cause deaths during the exposures or within 24 h following the exposures, since post-exposure deaths beyond this time are much less reproducible.

This report documents the research and development of a SRM for calibration of the overall test procedure and to assure that the cup furnace smoke toxicity apparatus is being used correctly. An acrylonitrile-butadiene-styrene (ABS) polymer whose characteristics fit the above criteria was selected. To perform this calibration, each laboratory would: 1. determine the *LC*_50_ values and compare them with the published certified *LC*_50_ values or 2. determine the N-Gas values (at the published certified *LC*_50_ values) and compare them with the certified N-Gas values. In the N-Gas approach, one or two animal tests are conducted at the certified *LC*_50_ values to assure that some percentage (not 0 and not 100%) of the animals respond. The calibration is conducted under two combustion modes (flaming and nonflaming) and two observation periods (a 30 min exposure and a 30 min exposure plus a 14 d post-exposure period). If the experimental values obtained by the laboratory fall within the 95% confidence intervals of the published certified values of this SRM, the investigator can be confident that the method is being conducted correctly.

## 2. Materials and Methods

### 2.1 Materials

A number of materials were screened before the decision to use an ABS polymer was made. The initial ABS that was selected for testing was standard reference material SRM 1007A used for calibrating the flaming mode in the ASTM E-662 and NFPA 258 test method to determine smoke density [9,10]. We found that this material exhibited suitable characteristics and fit the above criteria. However, during the experimental phase of this study, the stock of SRM 1007A was depleted. To conserve expense, the decision was made to obtain an ABS material suitable for SRMs for both the cup furnace smoke toxicity procedure and the smoke density chamber.

Three different ABS samples (ABS 2, ABS 3, and ABS 4) with formulations believed to be similar to SRM 1007A were sequentially prepared by a commercial manufacturer and tested in both the toxicity and smoke density apparatuses. Only ABS 4 proved to be suitable for both systems, and the manufacturer was asked to prepare a production lot, designated ABS 5 in this paper, for certification and sale as an SRM. ABS 4 and ABS 5 were thus presumably the same. In addition to acrylonitrile-butadiene-styrene, formulations 4 and 5 contained 6% titanium dioxide by weight. The ABS 5 was prepared by the manufacturer in sheets that were 254 × 254 mm (10 × 10 in) with a measured nominal thickness of 0.762 mm (0.030 in). The thickness was an important factor for the ASTM E-662 and NFPA 258 Smoke Density Chamber tests. The sheets of the production lot of ABS 5 were randomly numbered when received by NIST. The final cup furnace smoke toxicity SRM certified for sale is designated SRM 1048.

### 2.2 Gases

The cup furnace smoke toxicity method includes the chemical analysis of the following gases: CO, CO_2_, HCN, O_2_, HCl, and HBr. (The latter two halogen gases should be monitored if the material is known or suspected of generating these gases when thermally decomposed. The ABS samples tested for this SRM do not generate any HCl or HBr.) If the instrumentation is available, the concentration of NO*_x_* (both NO and NO_2_) may also be measured when nitrogen-containing materials, such as ABS, are being tested.

The calibration gases (CO, CO_2_, HCN) utilized in the research and development of this SRM were commercially supplied in various concentrations in nitrogen. The concentrations of HCN in the commercially supplied cylinders were routinely checked by silver nitrate titration [11], since it is known that the concentration of HCN stored under these conditions will decrease with time. Nitric oxide (NO) in nitrogen, a standard reference material, was obtained from the Gas and Particulate Science Division, NIST.

Carbon monoxide and carbon dioxide were measured continuously during each test by non-dispersive infrared analyzers. Oxygen concentrations were measured continuously with a paramagnetic analyzer. Syringe samples (100 μl) of the chamber atmosphere were analyzed for HCN approximately every 3 min with a gas Chromatograph equipped with a thermionic detector [12]. The concentration of NQ*_x_* was measured continuously by a chemiluminescent NO*_x_* analyzer with a sampling rate of 25 ml/min. All combustion products and gases (except HCN and NQ*_x_*) that were removed for chemical analysis were returned to the chamber. The CO, CO_2_,O_2_, and NO*_x_* data were recorded by an on-line computer every 15 s.

The presence of HCN in the combustion atmospheres interfered with the NO*_x_* analysis when the chemiluminescent analyzer was equipped with a stainless steel converter. A change of the stainless steel converter to a molybdenum (Mo) converter (set at 375 °C) prevented this interference from HCN. The amounts of NO and NO_2_ in the NO*_x_* can be distinguished by allowing the sample gas to pass through the converter (gives results for total NO*_x_*) or to bypass the converter (gives only NO results). The amount of NO_2_ is calculated from the difference of the two signals.

For each experiment, the reported gas concentrations are the time-integrated average exposure values which were calculated by integrating the area under the instrument response curve and dividing by the exposure time (i.e., (ppm × min)/min or, in the case of O_2_, (% × min)/min). The calculated CO and CO_2_ concentrations are accurate to within 100 and 500 ppm, respectively. The calculated HCN concentrations are accurate to 10% of the HCN concentration. The calculated NO*_x_* concentrations are accurate to 10% of the NO*_x_* concentration.

### 2.3 Animals

Fischer 344 male rats, weighing 200–300 g, were obtained from Taconic Farms (Germantown, NY).^1^ They were allowed to acclimate to our laboratory conditions for at least 10 d prior to experimentation. Animal care and maintenance were performed in accordance with the procedures outlined in the National Institutes of Health’s “Guide for the Care and Use of Laboratory Animals.” Each rat was housed individually in suspended stainless steel cages and provided with food (Ralston Purina Rat Chow 5012) and water *ad libitum.* Twelve hours of fluorescent lighting per day were provided using an automatic timer.

### 2.4 Cup Furnace Smoke Toxicity Procedure

**Fig. 1:**
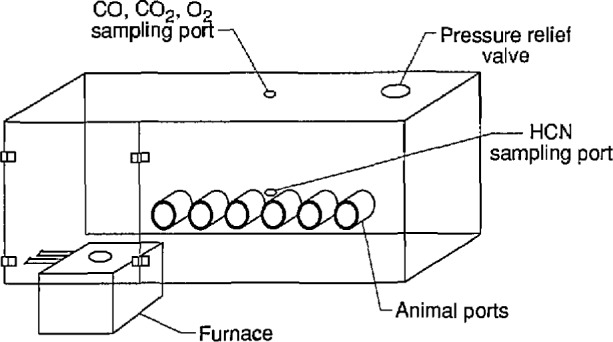
Cup furnace smoke toxicity exposure chamber.

**Fig. 2:**
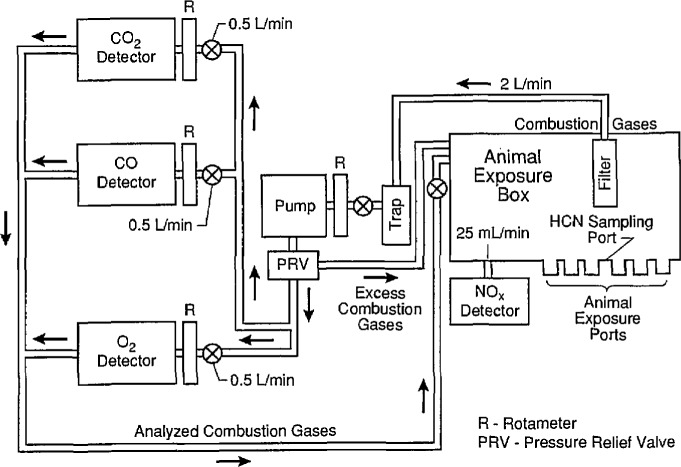
Schematic of cup furnace smoke toxicity exposure chamber with attached analytical equipment.

**Fig. 3:**
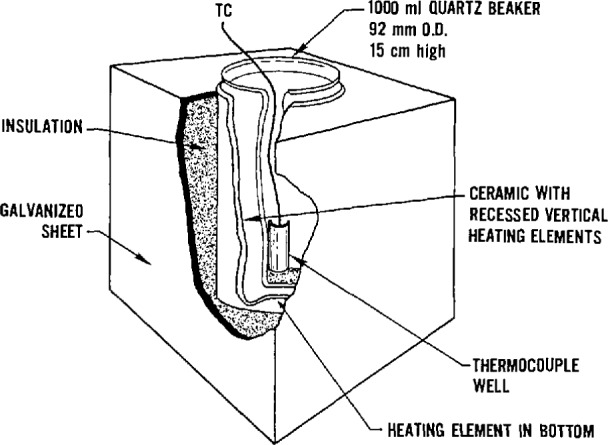
Cup furnace.

All exposures were conducted using the combustion system, the chemical analysis system, and the animal exposure system that were designed for the cup furnace smoke toxicity method [4]. Figures 1 and 2 are a diagram and schematic drawing of the experimental arrangement, respectively. The cup furnace is shown in Fig. 3. The samples were decomposed in the cup furnace located directly below the animal exposure chamber such that all the combustion products from the test sample evolved directly into the chamber. To prepare the test samples, the ABS sheets were cut into pieces approximately 2.54 cm^2^ (1 in^2^). Multiple squares were used to obtain the desired test concentration (defined as grams of material placed into the furnace divided by the exposure chamber volume in meters, i.e., g/m^3^ or mg/l).

Tests were conducted in both flaming and non-flaming modes. The autoignition temperature of ABS was determined according to the procedure described in the cup furnace smoke toxicity method [4] and the furnace was set approximately 25 °C below or above this autoignition temperature for the nonflaming or flaming modes, respectively. In the flaming tests, a sparker was also used to ensure that the ABS sample would flame as early as possible. This sparker was not used in the determination of the autoignition temperature.

The cup furnace smoke toxicity method is a closed design in which all the gases and smoke are kept in a 200 L rectangular chamber for the duration of the experiment. Six rats are exposed in each experiment. Each animal is placed in a restrainer and inserted into one of six portholes located along the front of the exposure chamber such that only the heads of the animals are exposed. In the experiments conducted to determine *LC*_50_ values, animal exposures started when the weighed sample was dropped into the preheated cup and continued for 30 min. The quartz cup which fits into the furnace and test specimen were weighed before and after the exposure to determine the mass of material consumed.

The toxicological endpoint was death, which occurred either during the 30 min exposures or the 30 min exposure plus 14 d post-exposure observation period.^2^ The percentage of animals dying at each fire effluent concentration was plotted to produce a concentration-response curve from which *LC*_50_ values were calculated for both the 30 min exposures and for the 30 min plus 14 d post-exposure observation period. The *LC*_50_ in these cases is defined as the mass of material placed in the furnace divided by the exposure chamber volume (g/m^3^) which caused 50% of the animals to die during the exposure only or during the exposure plus the 14 d post-exposure observation period. The *LC*_50_ values and their 95% confidence limits shown in Tables 2 through 6 were calculated by the statistical method of Litchfield and Wilcoxon [13]. The *LC*_50_ values provided in Tables 7 and 8 were calculated using probit analysis as described in Finney [14]. All animals (including the controls) were weighed daily from the day of arrival until the end of the 14 d post-exposure observation period.

### 2.5 N-Gas Model Prediction

The current N-Gas Model [15–18] equation is based on the studies at NIST of the toxicological interactions of six gases, CO, CO_2_, HCN, reduced O_2_, HCl, and HBr, and is used to estimate the amount of material (either loaded or consumed) necessary to produce an *LC*_50_ for a 30 min exposure or a 30 min exposure plus a 14 d post-exposure period. *LC*_50_ values for other exposure times can also be used. The model prediction is based on the following empirical mathematical relationship:

$$\begin{array}{l} \text{N-Gas Value}=\frac{m[\text{CO}]}{[{\text{CO}}_{2}]-b}+\frac{\left[\text{HCN}\right]}{LC_{50}\text{HCN}}\\ +\frac{21-[O_{2}]}{21-LC_{50}\;O_{2}}+\frac{\left[\text{HCl}\right]}{LC_{50}\text{HCl}}+\frac{\left[\text{HBr}\right]}{LC_{50}\text{HBr}} \end{array}$$ (1)

where the numbers in brackets are time-integrated average atmospheric concentrations during a 30 min exposure period [(ppm × min)/min or for O_2_ (% × min)/min]. We have found that CO_2_ acts synergistically with all toxic gases tested to date. However, empirically, we found that the CO_2_ term can be used in the equation only once. Therefore, the CO_2_ effect is utilized with the CO factor since CO is found in all fires and we have the most data on the CO and CO_2_ synergism [19]. As the concentration of CO_2_ increases [up to 50,000 ppm (5%)], the toxicity of CO increases. Above 50,000 ppm, the toxicity of CO starts to decrease again. The terms *m* and *b* define this synergistic interaction and equal −18 and 122000, if the CO_2_ concentrations are 50,000 ppm or less. For studies in which the CO_2_ concentrations are above 50,000 ppm, *m* and *b* equal 23 and −38600, respectively. The *LC*_50_ concentration of HCN is 200 ppm for 30 min exposures or 150 ppm for 30 min exposures plus 14 d post-exposure deaths. The 30 min exposure with or without the 14 d post-exposure *LC*_50_ value for O_2_ is 5.4%. Ideally, when this equation is unity, 50% of the animals should die. Examination of our animal lethality data for the three and four gas combinations indicate that the mean N-Gas value where animal deaths occur is 1.1 with a standard deviation of ± 0.1. We have found in the pure gas work that one half of the animals are likely to die when the N-Gas value is approximately 1.1, no animals usually die below 0.9 and all the animals usually die above 1.3.

The N-Gas Model has been developed into an N-Gas Method for predicting the concentration of material which would produce an *LC*_50_ [15,16]. This method reduces the time necessary to evaluate a material and the number of test animals needed for the toxic potency determination. It also indicates whether the toxicity is usual (i.e., the toxicity can be explained by the measured gases) or is unusual (i.e., additional gases are needed to explain the toxicity). The N-Gas approach has been shown to work well under different combustion systems (radiant as well as convective heat sources; bench-scale as well as full-scale room tests) [20–23].

To measure the toxic potency of a given material with this N-Gas Method, a sample is combusted under the conditions of concern (e.g., nonflaming or flaming) and the principal gaseous components (CO, CO_2_, HCN, reduced O_2_, HCl, and HBr) of the smoke measured. Based on the results of the chemical analytical tests and the knowledge of the interactions of the measured gases, an estimated *LC*_50_ value is calculated. If the N-Gas approach is to be used as a screening test, then in one or two further tests, six rats are exposed to the smoke from a sample of such size that the smoke should produce an atmosphere in which the N-Gas value would be less than or equivalent to 0.8. The deaths of some of the animals indicates the presence of one or more unknown toxicants. If more accuracy is needed, a detailed *LC*_50_ can be determined. An N-Gas value (at the *LC*_50_) above 1.3 suggests that a toxicological antagonism is occurring.

The screening test, however, is not appropriate if one wants to use the N-Gas approach with the ABS SRM 1048 to calibrate the cup furnace smoke toxicity method. In this case, N-Gas values equivalent to the actual *LC*_50_s for the ABS SRM are provided in the SRM certificate. A sample mass equal to the certified *LC*_50_ value is combusted under the conditions of concern (e.g., nonflaming or flaming) and the principal gaseous components (CO, CO_2_, HCN, and reduced O_2_) of the ABS smoke measured. Equation (1) is then used to determine if this mass of material produced the gas concentrations necessary to achieve N-Gas values equivalent to those listed on the certificate. Finding N-Gas values within the 95% confidence limits of the certified values indicates the same concentration of material decomposes to produce a similar chemical atmosphere. To test if the toxicity is correct, the same mass of material (i.e., equal to the certified *LC*_50_) is now used in one or two animal tests (N-Gas values are determined for these tests, too) in which the deaths of some percentage of the animals (not 0 and not 100%) indicates that the results of the laboratory are close to that of the certified SRM. Four N-Gas values (i.e., flaming, 30 min exposure; flaming, 30 min exposure plus 14 d post-exposure observation period; nonflaming, 30 min exposure; nonflaming, 30 min exposure plus 14 d post-exposure observation period) and their equivalent *LC*_50_ values are provided on the certificate and in Table 8. If the values found by the investigator fall within the 95% confidence limits of the certified values, the equipment can be assumed to be working correctly.

### 2.6 Comparison Factors in the Development of this SRM

#### 2.6.1 Autoignition Temperatures

In the intralaboratory evaluation of the various ABS formulations, the autoignition temperatures were independently determined for each formulation and before each new series of experiments designed to determine an *LC*_50_ value. Autoignition temperatures were also determined for ABS SRM 1007A by each of the participants in the interlaboratory evaluation.

#### 2.6.2 Interlaboratory Evaluation In

the process of selecting the SRM, it was necessary to examine the reproducibility of results across laboratories using a comparable material. Therefore, three laboratories (in addition to NIST) were asked to participate in an interlaboratory evaluation of ABS 1007A using the cup furnace smoke toxicity method. The laboratories which tested this material were Mobay (Stilwell, KS), NIST (Gaithersburg, MD), Southwest Research Institute (San Antonio, TX), and U.S. Testing (Hoboken, NJ). They agreed to determine the autoignition temperatures and *LC*_50_ values (30 min exposures and 14 d post-exposure observation period) for both the flaming and nonflaming modes. The interlaboratory evaluation was designed and conducted before we realized that the supply of ABS 1007A was limited. Since the interlaboratory results on ABS 1007A showed good reproducibility and ABS 1007A and ABS 5 were considered comparable materials, an additional interlaboratory evaluation of ABS 5 was not considered necessary.

#### 2.6.3 Intralaboratory Comparison

NIST examined the repeatability of the *LC*_50_ values (for both within the 30 min exposures and for within the 30 min exposures plus the 14 d post-exposure observation period). Enough tests were conducted to calculate three separate *LC*_50_ values for each of the flaming and nonflaming modes of ABS 5 (the final selected SRM), two *LC*_50_ values for each of the flaming and nonflaming modes of ABS sample 1007A, two *LC*_50_ values for the flaming mode of ABS 4, one *LC*_50_ value for the nonflaming mode of ABS 4, and one *LC*_50_ value for the nonflaming mode of ABS 3. Since ABS 2 and 3 were found to be unsuitable for the Smoke Density Chamber, complete *LC*_50_ values were not determined for every combustion mode.

#### 2.6.4 N-Gas Values

In the development of this SRM, both *LC*_50_s and N-Gas values were obtained for each series of experiments. N-gas prediction values at the *LC*_50_ concentrations were calculated as follows: first, the N-Gas value was determined for each experiment using Eq. (1). Then these N-gas values were plotted against their respective mass loading/chamber volumes. The best fit to the points was obtained by a least squares linear regression analysis. The N-Gas value at the *LC*_50_ was then determined from the mass loading/chamber volume equivalent to the experimentally determined *LC*_50_.

#### 2.6.5 Statistical Analysis

All of the data from the 71 experiments that were conducted with ABS 5 were submitted to the Statistical Engineering Division in the Computing and Applied Mathematics Laboratory at NIST. The following measurements for each experiment were examined: the concentration of smoke [i.e., mass loading/chamber volume (g/m^3^)] in the chamber, the number of rats that died during each 30 min exposure, the total number of rats that died during the 30 min exposures plus the post-exposure period of 14 d,^3^ and the N-Gas values for the 30 min exposures and for the 30 min exposures plus the post-exposure period. Although more chemical analytical data was available, the “summary statistic” of the N-Gas values was sufficient to meet the goals of the analysis. The 30 min within-exposure and the 30 min within exposure plus post-exposure data were analyzed separately for both the flaming and nonflaming experiments.

Probit analysis as described in Finney [14] was used to determine the *LC*_50_ values, the concentration at which 50% of the animals in such an experiment should die. Individual fits were done for each of the three series of experiments and the *LC*_50_ values for each series was determined. N-Gas computations were also done on a series-by-series basis. A straight line through the origin was fit to the N-Gas values as a function of the concentration (i.e., mass of material loaded into the furnace per chamber volume) for each series. Then the N-Gas value at the *LC*_50_ for that series was calculated. Thus, three observations (one for each series) of the N-Gas value at the *LC*_50_ for each combustion mode and observation period were obtained.

## 3. Results

### 3.1 Autoignition Temperature

The autoignition temperatures were determined for each tested formulation of ABS and three times for ABS 5 (once before each of the multiple series of tests on ABS 5) to examine the within laboratory repeatability (Table 1). Reproducibility between laboratories was tested only with ABS 1007A (Table 1). The interlaboratory evaluation was completed before we realized the stock of ABS SRM 1007A was limited.

In the interlaboratory evaluation of ABS 1007A, NIST found an autoignition temperature of 550 °C which was the same as that found by NIST for all the other ABS samples except #2 (see Table 1). The other laboratories, however, found autoignition temperatures ranging from 500 °C to 544 °C for ABS 1007A. Although the autoignition temperatures were different, the *LC*_50_ values that were determined by the other laboratories were in the same range (except for one laboratory in the flaming combustion mode) (see Table 2). The reasons for the differences in autoignition temperatures from the separate laboratories are unknown, but may be due to variations in furnace design, thermocouple placement, or the reference voltage of the thermocouple. Since the experiments are conducted at 25 °C above and below the autoignition temperature of the SRM (i.e., the temperature of the experiments are normalized by the material), comparable toxicological data were obtained. This aspect of the interlaboratory evaluation indicated that the autoignition temperature should not be one of the certified values of this SRM, but rather each user should determine their own autoignition temperature of the SRM according to the procedure specified in Ref. [4]. In other words, the SRM should be tested in the flaming and nonflaming modes which are, respectively, 25 °C above and below the autoignition temperature individually determined by each laboratory.

### 3.2 Interlaboratory Evaluation

All the toxicological and chemical data provided by the participants in the interlaboratory evaluation of ABS 1007A were analyzed by NIST and the *LC*_50_S, N-Gas values, and gas concentrations at the calculated *LC*_50_s are given in Tables 2, 3, and 4. Each of these values are the result of multiple experiments.

This evaluation of SRM 1007A showed (with one exception) that there was good reproducibility of results across laboratories (i.e., the *LC*_50_ values from the different laboratories were within the 95% confidence limits of the other laboratories). These results agree with our prior and much more extensive interlaboratory evaluation that was conducted on the cup furnace smoke toxicity method [5]. Although this interlaboratory evaluation was conducted with ABS SRM 1007A prior to the realization that the supply was limited, it was not considered necessary to repeat the interlaboratory evaluation with the new material, since the new material chosen to replace ABS SRM 1007A was designed to have a similar formulation.

### 3.3 Intralaboratory Evaluation

Six series of experiments were conducted at NIST on ABS 5 to examine the repeatability of results. Three series were in the nonflaming combustion mode and three were in the flaming combustion mode. Each series consisted of multiple experiments (designated by (n) in Tables 5 and 6). The *LC*_50_values were determined for each series for the deaths occurring during the 30 min exposures and for the deaths that occurred during the 30 min exposures plus the 14 d post-exposure observation period. The within-exposure results are given in Table 5 and the within plus post-exposure results are given in Table 6. The chemical analytical results for each gas were plotted against the concentration of material loaded into the furnace [mass loading/chamber volume (g/m^3^)] and the gas concentrations at the *LC*_50_ values were determined by a least squares linear regression analysis of the data. Tables 5 and 6 provide the calculated *LC*_50_ values, the calculated gas concentrations at the *LC*_50_s, and two sets of N-Gas values at the *LC*_50_s; one set of N-Gas values was determined from a least squares linear regression analysis of the data from the individual experiments and the other set of N-Gas values was calculated from the gas concentrations presented in Tables 5 and 6. The data shown in Tables 5 and 6 indicate the good repeatability of results obtained with ABS 5.

### 3.4 Statistical Analysis

**Fig. 4:**
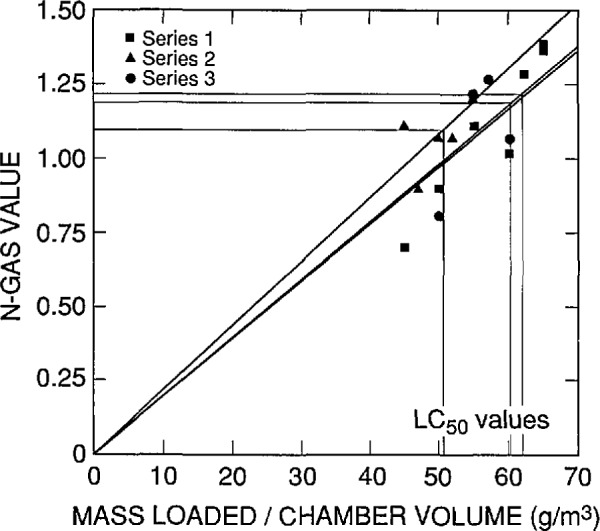
N-Gas values as a function of concentration [i.e., mass of material loaded into the cup furnace divided by the exposure chamber volume (g/m^3^)] for the three separate series of tests on ABS 5. Nonflaming mode, within exposure effects.

The Computing and Applied Mathematics Laboratory conducted a statistical analysis of the data from ABS 5 which is to be certified and sold by the Standard Reference Materials Program. Individual probit analysis fits were done for each series of experiments. (Three series were conducted to examine the repeatability of the *LC*_50_ values.) The *LC*_50_ value was calculated for each series, resulting in three observations of the *LC*_50_ for each combustion mode and observation time. N-Gas computations were also done on a series-by-series basis. It was important to examine the data on a series-by-series basis since there appeared to be systematic differences between the series for both the probits and the N-Gas values. For example, Fig. 4, which shows the three series of experiments for the within exposures to the nonflaming combustion mode, indicates that one series has a different relationship between the N-Gas values and the mass loading/chamber volume than the other two series (this can be seen by the fact that the slopes of the fitted lines are different for each series). If such systematic differences exist between series, one grand fit to all the data for a given mode and observation period might produce a biased estimate of the *LC*_50_ value or the corresponding N-Gas value.

The results of this statistical analysis are presented in Table 7 and in Figs. 4 through 7. In these figures, a straight line through the origin was fit by least squares linear regression analysis of the N-Gas values as a function to the mass loading/chamber volume for each series. Then the N-Gas value at the *LC*_50_ value for that series was determined resulting in three observations of the N-Gas value at the *LC*_50_ (one for each series) for each combustion mode and observation period.

The variation in the three observations incorporates both the uncertainty with each fit and the differences between series. Therefore, the mean and a confidence interval for the mean based on the three observations for each combustion mode and observation period summarize the *LC*_50_ and N-Gas values, giving the user of the SRM our best estimates of the true values for the material and how well we know them (Table 8). The intervals provided in Table 8 are the 95% confidence intervals based on two degrees of freedom. The *LC*_50_ and N-Gas values shown in Table 8 are the values that will be provided on the SRM certificate.

## 4. Discussion

An acrylonitrile-butadiene-styrene (ABS) has been evaluated and submitted for certification for use as a standard reference material (SRM 1048) for the cup furnace smoke toxicity method. An interlaboratory evaluation conducted by four laboratories on a comparable ABS material indicated good reproducibility of *LC*_50_ values (with one exception in the flaming mode) and N-Gas values across laboratories. This interlaboratory evaluation showed that the determination of the autoignition temperature of the test material was variable, but that if the experiments are conducted 25 °C above (flaming) and below (nonflaming) the individually determined autoignition temperatures, the chemistry and toxicity results were comparable between laboratories. These results indicate that the autoignition temperature should be determined by each laboratory and should not be included in the certified values of the SRM. In other words, the temperatures at which the experiments are conducted are normalized by the material and not by the temperature reading which could vary due to furnace construction, thermocouple placement or other differences between laboratory equipment.

Both the mean *LC*_50_ values and the N-Gas values ± their respective 95% confidence limits are provided in Table 8 and can be used with this SRM to calibrate the method and assure the user that the data that they obtained with this procedure is within the expected bounds. Since the N-Gas values were determined at the *LC*_50_s, the N-Gas values can be used instead of determining the complete *LC*_50_ value for each combustion mode (flaming or nonflaming) and observation period (within the exposure or within plus the post-exposure period). Utilization of the N-Gas values rather than determination of each *LC*_50_ value for comparison with the certified *LC*_50_ values has the advantages of reducing the number of needed experimental animals, the time necessary to complete the calibration tests, and the expense.

It is left to the user’s discretion whether complete *LC*_50_ values should be determined or if the N-Gas approach should be used. We recommend the latter approach. In the N-Gas approach, both the chemical (N-Gas values) and toxicological results (actual lethalities at the certified *LC*_50_ values) are compared to the certified values. To use the N-Gas approach, one needs to decompose the SRM at the certified *LC*_50_ values in either the flaming or nonflaming mode, measure the concentrations of pertinent gases, namely, CO, CO_2_, HCN, and O_2_, and determine the N-Gas value. Comparison of this value with the N-Gas value provided in the SRM certificate will show if the chemical results agree with the certified results. To determine if the toxicological results are comparable, the mass of material equivalent to the certified *LC*_50_ is decomposed in the presence of the rats as described in Ref. [4]. One or two experiments should indicate if the animals respond as expected (i.e., two to five rats die either within the 30 min exposure or within the 30 min exposure plus the 14 d post-exposure period, depending on which observation period is of interest).

It should be noted that with this particular material, the N-Gas values at the *LC*_50_ values are higher than unity, especially in the flaming mode. N-Gas values lower than unity indicate that toxic gases other than CO, CO_2_, HCN, and O_2_ may be contributing to the toxic atmospheres (i.e., making the combustion atmosphere more toxic than predicted). N-Gas values higher than unity indicate that one or more gases may be acting as a toxicological antagonist (i.e., making the combustion atmosphere less toxic than predicted). In our studies at NIST, we have found N-Gas values are higher than expected in those cases where the material produces a significant amount of HCN. Our recent data (to be published) indicates that in these cases, NO*_x_* is also formed. As expected, NO*_x_* was found in the combustion atmospheres of the ABS tested for this SRM (Tables 5 and 6). Our studies with NO_2_ indicate that exposure to NO_2_ increases the methemoglobin levels in the blood [24]. It is well known that methemoglobin acts as an antidote for cyanide poisoning by binding the CN^−^ and preventing it from being transferred to the tissues where the toxic insult occurs. We believe, therefore, that the N-Gas values at the *LC*_50_ values of this ABS are higher than expected because NO*_x_* causes the formation of methemoglobin which acts as an antidote for the HCN (i.e., an antagonistic effect occurs). An N-Gas equation including NO_2_ is being tested, but for the purposes of the use of this SRM is not necessary. The user can employ the certified N-Gas values and thus, will not be required to monitor NO*_x_* which requires additional analytical equipment that might not be readily available in many laboratories.

With SRM 1048, an investigator can calibrate both the chemical (based on the certified N-Gas values) and toxicological results (based on the certified N-Gas or *LC*_50_ values) from two combustion modes (flaming and nonflaming) in the cup furnace smoke toxicity method. If the experimental values fall within the 95% confidence limits of the certified values of this SRM, investigators can be confident that they are using the equipment properly.

## 5. Conclusions

A standard reference material ABS SRM 1048 has been developed to calibrate the cup furnace smoke toxicity method. The SRM material chosen is an acrylonitrile-butadiene-styrene (ABS) which is the same material used for SRM 1007B that has been recently certified for calibration of the flaming mode of the ASTM E-662 and NFPA 258 Smoke Density Chamber methods. Certified values plus their 95% confidence limits are provided for both the *LC*_50_ values and the N-Gas values for two combustion modes (flaming and nonflaming) and two observation periods (within the 30 min exposure or within the 30 min exposure plus a 14 d postexposure period). The certified *LC*_50_ values plus 95% confidence intervals (in g/m^3^) are 27 ± 3 (30 min, flaming); 25 ± 3 (30 min + 14 d, flaming); 58 ± 15 (30 min, nonflaming); and 53 ± 12 (30 min + 14 d, nonflaming). The certified N-Gas values plus 95% confidence intervals are 1.4 ± 0.2 (30 min, flaming); 1.5 ± 0.2 (30 min + 14 d, flaming); 1.2 ± 0.2 (30 min, nonflaming); and 1.4 ± 0.2 (30 min + 14 d; nonflaming). It is recommended that the users conserve experimental animals, time and expense by using the N-Gas approach to calibrate their system rather than conducting the complete determination of the *LC*_50_ values.

## Figures and Tables

**Fig. 5:**
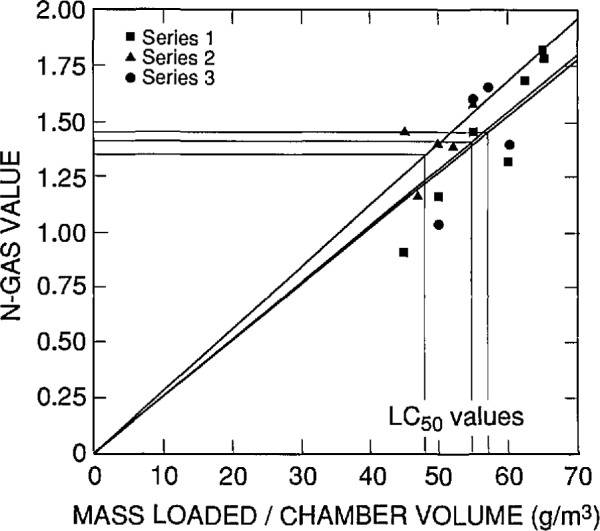
N-Gas values as a function of concentration [i.e., mass of material loaded into the cup furnace divided by the exposure chamber volume (g/m^3^)] for the three separate series of tests on ABS 5. Nonflaming mode, within plus post-exposure effects.

**Fig. 6:**
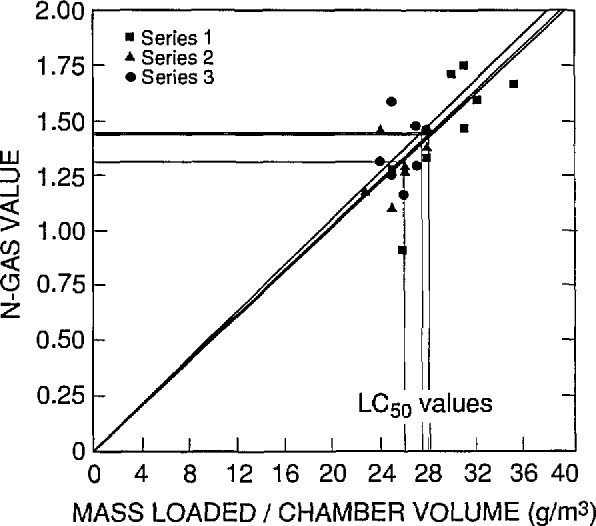
N-Gas values as a function of concentration [i.e., mass of material loaded into the cup furnace divided by the exposure chamber volume (g/m^3^)] for the three separate series of tests on ABS 5. Flaming mode, within exposure effects.

**Fig. 7:**
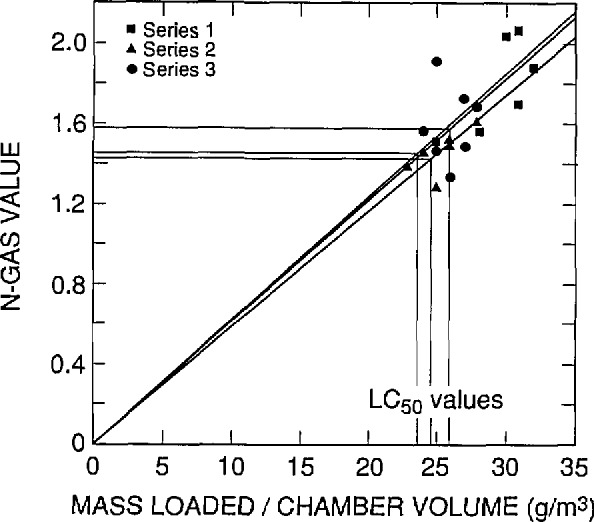
N-Gas values as a function of concentration [i.e., mass of material loaded into the cup furnace divided by the exposure chamber volume (g/m^3^)] for the three separate series of tests on ABS 5. Flaming mode, within plus post-exposure effects.

**Table 1:** Autoignition temperatures

ABS designation	Laboratory	Autoignition temperatures (°C)		
		Series 1^a^	Series 2^a^	Series 3^a^
1007A	NIST	550		
1007A	#4	532–544		
1007A	#5	515		
1007A	#3	500		
ABS 2	NIST	575^b^		
ABS 3	NIST	550		
ABS 4	NIST	550		
ABS 5	NIST	550	550	550

a When the final ABS formulation was chosen, three series of separate experiments were conducted to examine the repeatability of results.

b In the determination of the autoignition temperatures, 1 g samples are tested to determine the temperature range. Then, an 8 g sample is tested to see if the higher loading will reduce the temperature. In the case of ABS 2, the amount of sample was limited, so the 8 g sample was not tested. ABS 2 proved to be unsuitable for the Smoke Density Chamber, so further testing was not pursued.

**Table 2:** Interlaboratory evaluation of ABS 1007A. Toxicological data

Laboratory	*LC*_50_ values^a^ (g/m^3^)	
	Nonflaming	Flaming
NIST #1	40 (33–49)^b^	25 (21–29)
NIST #2	37 (32–43)	26 (24–29)
Laboratory #3	34 (24–47)	≈25^c^ [22.5^d^–25^e^]
Laboratory #4	29 (25–33)	26 (23–30)
Laboratory #5	38^f^(33–43)	41^f^(38–44)

a Calculated based on deaths within the 30 min exposure plus the 14 d post-exposure observation period.

b 95% confidence limits, computed using the method of Litchfield and Wilcoxon [13].

c Estimated from range of values, see footnotes d and e.

d No animals died at this concentration.

e Five out of the six exposed animals died at this concentration.

f These values were calculated at NIST in same manner as all other values in this table. Calculations by Laboratory # 5 resulted in slightly lower values. (Nonflaming was 34 g/m^3^ with 95% confidence limits of 30–38; flaming was 38 g/m^3^ with 95% confidence limits of 32–41.)

**Table 3:** Interlaboratory evaluation of ABS 1007A. Nonflaming mode chemical data^a^

Lab #	*LC*_50_^b^ (g/m^3^)	CO (ppm)	CO_2_ (ppm)	HCN (ppm)	O_2_ (%)	N-Gas value
NIST #1	40 (33–49)^c^	450 (370–550)^d^	2960 (2610–3410)	170 (140–200)	20.5 (20.5–20.4)	1.2^e^ (1.0–1.5) 1.2^f^
NIST #2	37 (32–43)	440 (380–580)	3800 (3340–4350)	180 (150–210)	20.4 (20.5–20.3)	1.3^e^ (1.1–1.5) 1.3^f^
Lab #3	34 (24–47)	NDP^h^	NDP	NDP	NDP	NDP
Lab #4	29 (25–33)	710 (610–800)	7410 (6520–8300)	150 (130–170)	20.0 (20.1–19.9)	1.2^e^ (1.1–1.3) 1.1^f^
Lab #5	38 (33–44)	420 (360–490)	2270 (2140–2440)	NM^i^	20.5 (20.5–20.4)	NC^j^

a Time-integrated average concentration over the 30 min exposure period calculated at the *LC*_50_ value. Based on the least squares analysis of the average 30 min gas concentrations at each mass loading tested.

b Based on deaths which occurred within- and post-exposure.

c 95% confidence limits on *LC*_50_ value, computed using the method of Litchfield and Wilcoxon [13].

d The gas concentrations calculated at the low and high 95% confidence limits of the *LC*_50_.

e Based on a least squares analysis of the N-Gas values for each experiment as a function of the mass loading. N-Gas value is that found at the *LC*_50_.

f Based on gas concentrations provided in this table.

h NDP—no data provided.

i NM—not measured.

j NC—not calculated due to lack of HCN data.

**Table 4:** Interlaboratory evaluation of ABS 1007A. Flaming mode chemical data^a^

Lab #	*LC*_50_^b^ (g/m^3^)	CO (ppm)	CO_2_ (ppm)	HCN (ppm)	O_2_ (%)	N-Gas value
NIST #1	25 (21–29)^c^	1600 (1300–1800)^d^	28800 (24500–33300)	120 (100–130)	17.3 (17.9–16.8)	1.2^e^ (0.9–1.5) 1.3^f^
NIST #2	26 (24–29)	1700 (1500–1900)	31100 (28800–34700)	110 (100–120)	17.0 (17.2–16.5)	1.3^e^ (1.1–1.4) 1.3^f^
Lab #3	25 (23–25)	NDP^h^	NDP	NDP	NDP	NDP
Lab #4	26 (23–30)	2200 (1900–2100)	36400 (3280–41200)	90 (80–100)	17.8 (18.1–17.4)	1.3^e^ (1.2–1.5) 1.3^f^
Lab #5	41 (38–44)	2500 (2300–2700)	42100 (39100–45000)	NM^i^	18.6 (18.7–18.4)	NC^j^

a Time-integrated average concentration over the 30 min exposure period calculated at the *LC*_50_ value. Based on the least squares analysis of the average 30 min gas concentrations at each mass loading tested.

b Based on deaths which occurred within- and post-exposure.

c 95% confidence limits on *LC*_50_ value, computed using the method of Litchfield and Wilcoxon [13].

d The gas concentrations calculated at the low and high 95% confidence limits of the LCso.

e Based on a least squares analysis of the N-Gas values for each experiment as a function of the mass loading. N-Gas value is that found at the *LC*_50_.

f Based on gas concentrations provided in this table.

h NDP—no data provided.

i NM—not measured.

j NC—not calculated due to lack of HCN data.

**Table 5:** Intralaboratory evaluation of ABS 5. Within-exposure NIST Chemical data^a^ at the *LC*_50_ value

Series *#* (n)^f^	*LC*_50_ (g/m^3^)	CO (ppm)	CO_2_ (ppm)	HCN (ppm)	O_2_ (%)	NO*_x_* (ppm)	NO (ppm)	NO_2_ (ppm)	N-Gas value
Nonflaming mode									

1 (6)	62(53–71)^b^	420(360–480)^c^	3490(3060–3910)^c^	220(190–250)^c^	20.4(20.5–20.3)^c^	NM^g^	NM	NM	1.2^d^(1.0–1.5) 1.2^e^
2 (5)	54(49–60)	420(380–470)	3800(3500–4170)	210(190–240)	20.3(20.4–20.3)	ND^h^	ND	ND	1.1(1.1–1.2) 1.2
3 (4)	60(56–64)	440(410–460)	3400(3210–3590)	220(200–230)	20.4(20.4–20.3)	NM	NM	NM	1.2(1.1–1.3) 1.2

Flaming mode									

1 (7)	29(28–31)	1900(1840–2030)	32200(31100–34300)	160(150–170)	16.7(16.8–16.4)	130(130–140)^e^	110(110–120)^e^	17(17–19)	1.5(1.4–1.6) 1.5
2 (6)	28(26–30)	1900(1760–2030)	34200(31800–36600)	150(140–160)	16.5(16.9–16.2)	120(120–130)	110(110–120)	18(16–19)	1.3(1.3–1.4) 1.4
3 (7)	27(26–28)	1780(1710–1840)	33700(32500–34900)	150(150–160)	16.5(16.7–16.3)	120(120–130)	100(95–100)	18(17–19)	1.4(1.4–1.4) 1.4

a Time-integrated average concentration over the 30 min exposure period calculated at the *LC*_50_ value. Based on the least squares analysis of the average 30 min gas concentrations at each mass loading tested.

b Values in parenthesis are the 95% confidence limits of the *LC*_50_ value.

c Values in parenthesis are the gas concentrations calculated at the low and high 95% confidence limits of the *LC*_50_s.

d Based on least squares analysis of N-Gas values at each of the mass loadings.

e Based on gas concentrations provided in this table.

f (n) – number of experiments in each series of tests.

g NM – not measured.

h ND – not detected based on two experiments.

**Table 6:** Intralaboratory evaluation of ABS 5. Within plus post-exposure NIST chemical data^a^ at the *LC*_50_

Series *#* (n)^f^	*LC*_50_ (g/m^3^)	CO (ppm)	CO_2_ (ppm)	HCN (ppm)	O_2_	NO*_x_* (ppm)	NO (ppm)	NO_2_ (ppm)	N-Gas value
Nonflaming mode									

1 (6)	60(55–66)^b^	410(370–450)^e^	3390(3160–3670)^c^	210(190–230)^c^	20.4(20.4–20.3)^c^	NM^g^	NM	NM	1.5^d^(1.3–1.8) 1.5^e^
2 (5)	50(48–53)	390(380–420)	3560(3440–3740)	200(190–210)	20.4(20.4–20.4)	ND^h^	ND	ND	1.4(1.3–1.4) 1.4
3 (4)	56(52–60)	410(380–440)	3210(3020–3400)	200(190–220)	20.4(20.5–20.4)	NM	NM	NM	1.4(1.3–1.6) 1.4

Flaming mode									

1 (7)	26(24–29)	1700(1570–1900)	29000(26800–32200)	140(130–160)	17.1(17.4–16.7)	120(110–130)^c^	100(90–110)^c^	16(14–17)^c^	1.6(1.5–1.7) 1.5
2 (6)	25(24–26)	1690(1630–1760)	30600(29400–31800)	130(130–140)	17.0(17.2–16.9)	110(110–120)	100(100–110)	16(15–16)	1.5(1.5–1.5) 1.5
3 (7)	25(23–27)	1640(1510–1770)	31200(28800–33700)	140(130–150)	16.9(17.2–16.5)	110(100–120)	90(80–100)	16(15–18)	1.6(1.6–1.6) 1.5

a Time-integrated average concentration over the 30 min exposure period calculated at the *LC*_50_ value. Based on the least squares analysis of the average 30 min gas concentrations at each mass loading tested.

b Values in parenthesis are the 95% confidence limits of the *LC*_50_ value.

c Values in parenthesis are the gas concentrations calculated at the low and high 95% confidence limits of the *LC*_50_s.

d Based on least squares analysis of N-Gas values at each of the mass loadings.

e Based on gas concentrations provided in this table.

f (n)–number of experiments in each series of tests.

g NM–not measured.

h ND–not detected based on two experiments.

**Table 7:** Statistical analysis of *LC*_50_ values and N-Gas values for ABS 5

Combustion mode	Observation time	Test series	*LC*_50_ values (g/m^3^)	N-Gas values
Flaming	WE^a^	1	28	1.4
		2	26	1.3
		3	28	1.4
	WE & PE^b^	1	26	1.6
		2	25	1.4
		3	24	1.5
Nonflaming	WE	1	62	1.2
		2	51	1.1
		3	60	1.2
	WE & PE	1	57	1.5
		2	48	1.3
		3	55	1.4

a WE–deaths occurred within the 30 min exposure.

b WE & PE–combined deaths that occurred either within the 30 min exposure and/or the 14 d post-exposure observation period.

**Table 8:** Mean *LC*_50_ and N-Gas values plus their 95% confidence limits for ABS 5

Observation time	Combustion mode	*LC*_50_ ± 95% CL^a^ (g/m^3^)	N-Gas value ± 95% CL
WE^b^	Flaming	27 ± 3	1.4 ± 0.2
	Nonflaming	58 ± 15	1.2 ± 0.2
WE & PE^c^	Flaming	25 ± 3	1.5 ± 0.2
	Nonflaming	53 ± 12	1.4 ± 0.2

a CL–95% confidence limits.

b WE–within the 30 min exposure.

c WE & PE–within the 30 min exposure plus the 14 d post-exposure observation period.

## Footnotes

1 Certain commercial equipment, instruments, or materials are identified in this paper to specify adequately the experimental procedure. Such identification does not imply recommendation or endorsement by the National Institute of Standards and Technology, nor does it imply that the materials or equipment identified are necessarily the best available for the purpose.

2 Under our experimental conditions, the rats exposed to the combustion products of ABS 5 died either during the exposure or within 24 h. However, we routinely use a 14 d post-exposure observation period. If animals were to die during the 13 d period after the first 24 h following exposure to the ABS 5 smoke, it may be indicative that something in addition to the smoke exposure was affecting the animals (e.g., the animals may be harboring an unknown pathogen). This could be important information to prevent misinterpretation of one’s data when similar deaths occur following exposures to the smoke from other materials.

3 All deaths occurred within the first 24 h following exposure, but surviving animals were kept and weighed for the full 14 d to assure no further deaths occurred.
